# "Egg shell” in bladder: A calculus around neglected Foley balloon catheter

**DOI:** 10.4103/0970-1591.65410

**Published:** 2010

**Authors:** Dharamveer Singh, Pawan Vasudeva, Apul Goel

**Affiliations:** Department of Urology, C.S.M. Medical University (Upgraded King George's Medical College), Lucknow-226 003, Uttar Pradesh, India

**Keywords:** Calculus, catheter, encrustation

## Abstract

Suprapubic catheterization of the bladder is used as a short- or long-term alternative to urethral catheterization. As with any indwelling urinary catheter, proper care is vitally important to minimize complications.

## INTRODUCTION

Suprapubic catheterization of the bladder is used as a short- or long-term alternative to urethral catheterization.[1] Long-term indwelling catheters are rarely completely free of complications such as bacteriuria, encrustation by mineral salts (40-50% of patients), pericatheter leakage, trauma, stone formation, balloon non-deflation, and catheter stuck while removal due to cuffing effect of deflated balloons.[1,2] We report one such case where a stone formed over the catheter giving the appearance of “egg-shell” in the bladder.

## CASE REPORT

**Figure 1:**
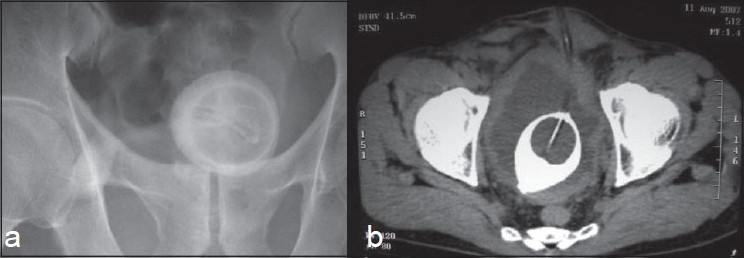
(a) X-ray pelvis shows a radiolucent core surrounded by calcification: “Egg Shell” calcification, (b) CT scan shows a calculus around the Foley balloon catheter

**Figure 2:**
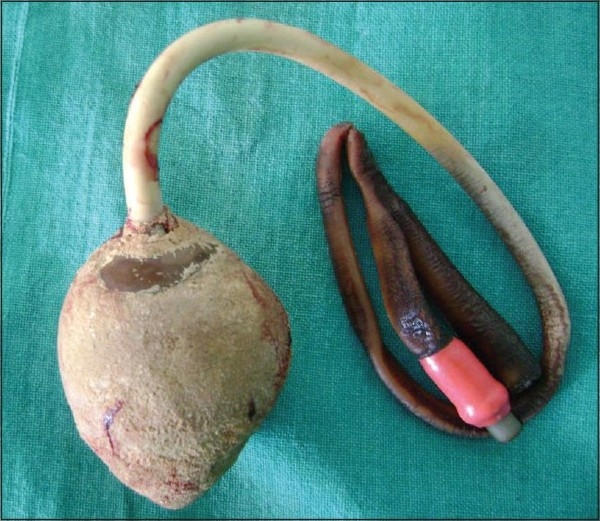
Calculus around the Foley catheter balloon, retrieved intact

A 55-year-old male underwent suprapubic catheter placement for urethral stricture two years ago. After a few months the catheter stopped draining and there was continuous pericatheter leakage of urine. Due to monetary constraints the patient did not seek medical attention for about 18-months and the suprapubic catheter remained unchanged over this period of time. When he presented to us, his serum creatinine was 2.5 mg%. Further evaluation, including ultrasonography, x-ray and CT scan pelvis revealed that a stone had formed over the catheter giving an appearance of “egg-shell' [Figure 1a and b]. He underwent open suprapubic cystolithotomy under spinal anesthesia with intact retrieval of stone around the Foley catheter balloon [Figure 2]. Subsequently, the urethral stricture was managed by buccal mucosa graft urethroplasty.

## DISCUSSION

Development of bacteriuria in the presence of an indwelling catheter is inevitable and the duration of catheterization is the most important risk factor for the development of bacteriuria which occurs at an incidence of approximately 10% per day of catheterization.[3] Risk of infection with a single catheterization is one to two per cent. In the bacteriuric urinary tract, there are two populations of bacteria: (1) planktonic growth, those bacteria growing in suspension in the urine and (2) biofilm growth (layers of organisms on infected indwelling catheters).[4] Bacteria attach to the catheter surface forming a biofilm (usually mixed communities of two or more micro-organisms) and secrete an extra-cellular polysaccharide matrix of bacterial glycocalices. The host urinary protein and salts complex with this matrix, leading to encrustation of the catheter lumen.[1,2] Colonization with urease-producing micro-organisms increases urinary pH (by converting urea into ammonia) which promotes precipitation of struvite (magnesium-ammonium-sulfate), and apatite (calcium-phosphate) crystals resulting in catheter encrustation and bladder stones.

Although some catheter materials are more resistant to encrustation than others, sooner or later, all catheters become encrusted. Elimination of urease-producing micro-organisms is required for reduction in urinary pH to prevent catheter encrustation. Laboratory-based evidence shows that filling the catheter-balloon with an antimicrobial-agent such as triclosan can prevent encrustation by the mixed flora of uropathogens like *Proteus mirabilis/vulgaris, Escherichia coli* and *Klebsiella pneumoniae* but had no effect on *Providencia rettgeri, Enterococcus faecalis and Pseudomonas aeruginosa*.[5] Acidic catheter maintenance solutions (or 'bladder washouts') can be effective in dissolving encrustations, thus reducing the need of frequent catheter change.[1,2]

Some urologists advocate the use of SPC rather than a urethral catheter to avoid the complications associated with long-term urethral catheters such as epididymo-orchitis, prostatitis, periurethral abscess, and pressure-effect complications (erosion of the bladder neck and urethra, urethral stricture, penile skin/prepucial necrosis). Technically, SPC is more demanding, so, when blockage or dislodgement occurs, nursing personnel may be reluctant to change the SPC without physician assistance.[6] One may face the problem of catheter stuck during catheter change/removal because of a 'cuffing' effect of the deflated balloon, especially with all-silicone catheters.[1]

Good catheter hygiene, including aseptic catheter insertion and sterile continuous closed drainage systems, is necessary to minimize the introduction of microorganisms into the bladder.[3] The catheter-meatal junction should be cleaned daily with water, but antimicrobial agents should be avoided because they lead to colonization with resistant pathogens. A three-weekly catheter change is advised by some to minimize encrustation. If the catheter is to be retained longer, periodic balloon deflation and reinflation to break overlying encrustations and bladder irrigation with an acidic solution is advised.[7]

Indwelling catheters should be avoided and wherever possible intermittent catheterization is a much better option. If an indwelling catheter is required, adequate catheter care should be instituted. Our case, a “sadhu,” did not seek medical attention despite constant pericatheter overflow consequent to a blocked catheter.
